# A Three-Gene Peripheral Blood Potential Diagnosis Signature for Acute Rejection in Renal Transplantation

**DOI:** 10.3389/fmolb.2021.661661

**Published:** 2021-05-04

**Authors:** Yicun Wang, Di Zhang, Xiaopeng Hu

**Affiliations:** ^1^ Department of Urology, Beijing Chao-Yang Hospital, Capital Medical University, Beijing, China; ^2^ Institute of Urology, Capital Medical University, Beijing, China

**Keywords:** renal transplantation, acute rejection, diagnostic signature, peripheral blood, gene expression, T-cell mediated rejection, immune cell analysis

## Abstract

**Background:** Acute rejection (AR) remains a major issue that negatively impacts long-term allograft survival in renal transplantation. The current study aims to apply machine learning methods to develop a non-invasive diagnostic test for AR based on gene signature in peripheral blood.

**Methods:** We collected blood gene expression profiles of 251 renal transplant patients with biopsy-proven renal status from three independent cohorts in the Gene Expression Omnibus database. After differential expression analysis and machine learning algorithms, selected biomarkers were applied to the least absolute shrinkage and selection operator (LASSO) logistic regression to construct a diagnostic model in the training cohort. The diagnostic ability of the model was further tested in validation cohorts. Gene set enrichment analysis and immune cell assessment were also conducted for further investigation.

**Results:** A novel diagnostic model based on three genes (*TSEN15, CAPRIN1* and *PRR34-AS1*) was constructed in the training cohort (AUC = 0.968) and successfully verified in the validation cohort (AUC = 0.925) with high accuracy. Moreover, the diagnostic model also showed a promising value in discriminating T cell-mediated rejection (TCMR) (AUC = 0.786). Functional enrichment analysis and immune cell evaluation demonstrated that the AR model was significantly correlated with adaptive immunity, especially T cell subsets and dendritic cells.

**Conclusion:** We identified and validated a novel three-gene diagnostic model with high accuracy for AR in renal transplant patients, and the model also performed well in distinguishing TCMR. The current study provided a promising tool to be used as a precise and cost-effective non-invasive test in clinical practice.

## Introduction

Over the last decades, although advances in kidney transplantation have resulted in remarkable improvements in graft survival, acute renal allograft rejection (AR) remains unavoidable with an incidence of approximately 7.8% among adult recipients (Hart et al., 2019). Besides, early occurrence of AR was found to be associated with increased risks of graft failure and death, particularly death from cancer and cardiovascular disease (Wu et al., 2015; Clayton et al., 2019). Therefore, AR is still a critical factor leading to the sub-optimal long-term outcomes of post-transplant patients (Meier-Kriesche et al., 2004; Lamb et al., 2011). These results suggest the necessity of timely and accurate diagnosis of AR in kidney transplant recipients, which may contribute to preserving renal function and improving consequences beyond the early period after transplantation.

Since many conditions other than AR may lead to renal allograft dysfunction, the diagnosis of AR cannot be made by monitoring insensitive functional indicators alone like serum creatine or urine protein (Li et al., 2012). Besides, approximately 10% of patients with clinically normal kidney function are found to have evidence of AR based on surveillance biopsy (Thierry et al., 2011). Currently, the gold standard for AR diagnosis still relies on obtaining kidney biopsies. It is one strategy to diagnose and treat AR before extensive injury by performing routine protocol biopsies. However, it is limited by procedural cost, assessment variability, the risk of infection and other stresses (Li et al., 2012). Researchers have reported a 1% incidence of major complications in large series and an increased risk of chronic rejection for renal transplants followed protocol biopsies (Schwarz et al., 2005; Moreso et al., 2012). Additionally, AR is such a dynamic process that is required to predict rejection and manage immunosuppression by minimally invasive monitoring not possible using biopsies. Thus, there is a pressing need to develop a less-invasive, convenient and accurate test for the diagnosis of AR.

Machine learning techniques, a specialization in statistics and computer science, focuses on how computers learn from data (Walsh et al., 2019). It is widely used for biological knowledge mining through analyzing large amounts of data on patient history, laboratory results, diagnoses and outcomes (Deo, 2015). In this study, we applied two advanced and commonly accepted algorithms, random forest (RF) and support vector machine-recursive feature elimination (SVM-RFE), to choose robust biomarkers for AR diagnosis in peripheral blood microarray datasets of AR patients from Gene Expression Omnibus (GEO) database. Moreover, the least absolute shrinkage and selection operator (LASSO) logistic regression was also applied to construct a streamlined diagnostic model that can be capable of extensively implementing in clinical practice. Although previous researches have reported several gene-based signatures for AR diagnosis, many signatures contain dozens or even hundreds of genes which limited their clinical translation (Friedewald et al., 2019; Cao et al., 2020), and their diagnostic ability was markedly reduced in other independent cohorts (Zhang et al., 2019; Cao et al., 2020). Therefore, based on machine learning methods, we aimed to create a novel peripheral diagnostic model of AR with minimal gene number, stable and satisfying performance.

## Materials and Methods

### Data Collection and Preprocessing

**FIGURE 1:**
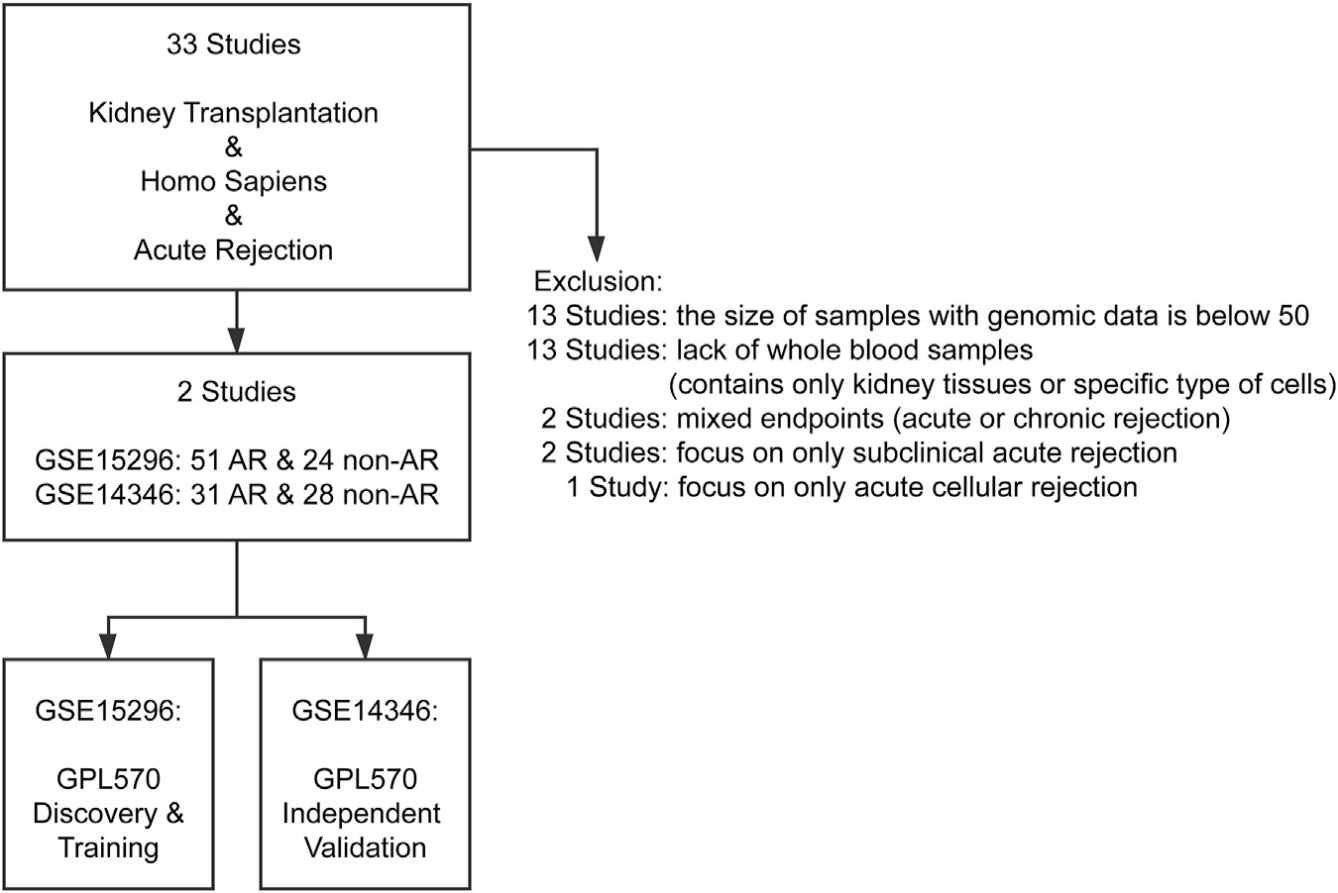
Flowchart of GEO datasets selection. After systematically screening the GEO database, two cohorts comprising gene expression profiles of more than 50 peripheral blood samples with biopsy-proven AR or non-AR were included for analysis. AR: acute rejection.

As demonstrated in Figure 1, thirty-three studies were initially included by using the search string “kidney transplantation”, “*Homo sapiens*” and “acute rejection” in the GEO database (https://www.ncbi.nlm.nih.gov/geo/). Subsequently, studies that met one of the following criteria were excluded: 1) number of samples with genomic data less than 50; 2) lacking whole blood samples with gene expression profiles; 3) focusing on other types of rejection, such as subclinical acute rejection. Finally, two independent studies with gene expression profiles of peripheral blood cells arising from corresponding kidney transplant patients with unequivocal biopsy-proven AR or non-AR were eligible for further analysis. The GSE15296 (*n* = 75) microarray dataset includes 51 AR and 24 non-AR samples, and the GSE14346 (*n* = 59) microarray dataset consists of 31 AR and 28 non-AR samples (Li et al., 2012; Kurian et al., 2014). Additionally, the GSE129166 (*n* = 117) was utilized for further detecting the ability of the diagnostic model in distinguishing T-cell mediated rejection (TCMR) and antibody-mediated rejection (ABMR) with 26 TCMR and 91 non-TCMR, 30 ABMR and 87 non-ABMR (Van Loon et al., 2019). The above three datasets were all based on the GPL570 platform (Affymetrix Human Genome U133 Plus 2). Microarray datasets were normalized and log_2_ transformed through “limma” R package, and then Z-score scaling was performed (Ritchie et al., 2015).

Data collection and preprocessing were fully conducted under GEO data access policies. All analyses were performed under relevant guidelines and regulations.

### Study Design

**FIGURE 2:**
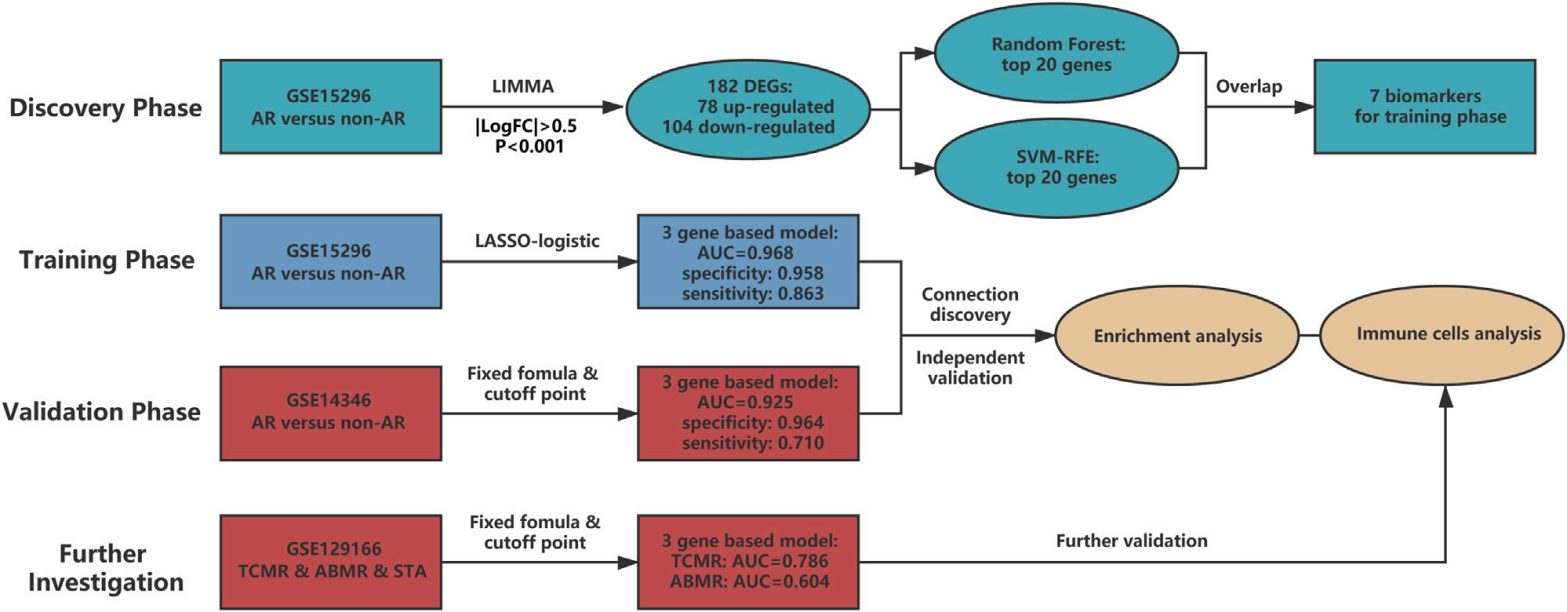
The main structure of current study. Four phases are included in our research. The discovery phase chose candidate biomarkers for AR, and then biomarkers were utilized to develop a three-gene based model in the training phase. Diagnostic ability of the model was deeply verified in the validation and further investigation phases. Gene set enrichment analysis and immune cell analysis were also conducted to detect potential mechanisms of AR. AUC: area under the curve. TCMR: T cell-mediated rejection. ABMR: antibody-mediated rejection. STA: stable. SVM-RFE: support vector machine - recursive feature elimination.

In the current research, we included four phases to identify and validate the gene-based peripheral blood diagnostic model for AR (Figure 2). In the discovery phase, GSE15296 was applied to screen differentially expressed genes (DEGs), followed by two machine learning approaches to select key biomarkers for AR. In the training phase, LASSO logistic regression was used to identify informative genes and construct a diagnostic model with low variance and strong universality. In the validation phase, the performance of the model was verified in GSE14346 which was derived from multi-centers. In the process for further investigation, the ability of the diagnostic model for assessment of TCMR and ABMR was tested in GSE129166. Besides, enrichment and immune-cell analysis were performed in all three datasets mentioned above to obtain a robust association between the model and overall immune status in renal transplant patients.

### DEGs Screening in AR

To filter out genes that specific to AR development, DEGs were identified utilizing the GSE15296 dataset through “limma” R package (Ritchie et al., 2015). The threshold was set as the absolute value of log_2_-fold change >0.5 and the adjusted *p*-value < 0.001.

### Gene Selection by Applying Machine Learning Methods

Two machine learning algorithms were implemented in our research to perform a binary classification (AR vs. non-AR). The RF algorithm is a supervised classification method based on an ensemble of decision trees that estimate the importance of features to distinguish samples with or without AR. Feature importance corresponded to the Gini importance measure was utilized to rank genes in the RF classifier by “randomForest” R package (Liaw and Wiener, 2002). SVM-RFE is a recursive feature elimination strategy, which utilizes the weighted vector produced by the classification model support vector machine. After being trained to optimize the classification accuracy between AR and non-AR samples, the SVM algorithm develops a weight vector for the input genes. The generated weight vector will be used to rank the genes, and the RFE strategy will recursively eliminate the least ranked genes. The R package “e1071” was used for SVM-RFE in this study (Guyon et al., 2002). Top-ranked genes selected simultaneously by RF and SVM-RFE were included in further analysis.

### Generation and Validation of the Diagnostic Model

During the process of LASSO logistic regression, the penalty regularization parameter lambda was chosen through 10-fold cross-validation by performing “glmnet” R package (Friedman et al., 2010). When minimal binomial deviance was set as the cross-validated condition, the genes with non-zero coefficients were picked out and formed the diagnostic model. The linear combination of the regression coefficient derived from the LASSO logistic regression model multiplied by gene expression level generated the diagnostic risk score. Receiver operating characteristic (ROC) curves were employed to measure the diagnostic performance and the optimal ROC cutoff value (Youden index) was computed through “pROC” R package, corresponding to the point of ROC curve where the sum of sensitivity and specificity for distinguishing AR and non-AR patients reached highest. Additionally, GSE14346 was utilized for external validation with fixed formula and the same cutoff point. Patients in both cohorts were divided into high- and low-risk groups with the same cutoff point.

### Assessment of TCMR and ABMR

Another independent cohort GSE129166 from a multicenter and prospective study was utilized to test the capability of the model in discriminating TCMR and ABMR with fixed formula and the same cutoff point. The ROC curves with AUC were displayed for evaluating its sensitivity and specificity.

### Functional Enrichment Analysis

Metascape (https://metascape.org/gp/index.html#/main/step 1) is a powerful annotation analysis tool for gene function by integrating several authoritative data resources (Zhou et al., 2019). Based on DEGs screened between AR and non-AR peripheral blood samples, Metascape was applied to analyze the potential signaling pathways occurring in the episodes of AR. The threshold was set as an adjusted *p*-value < 0.05, a minimum overlap of five genes, and a minimum enrichment score of two. Besides, to further detect potential biological processes enriched in the high-risk group, gene set enrichment analysis (GSEA) was performed by applying “clusterProfiler” R package annotated by reference gene set file (c5. bp.v7.0. entrez.gmt) (Yu et al., 2012).

### Evaluation of Immune Cells

The amounts of immune cell subtypes were quantified by single sample gene set enrichment analysis (ssGSEA) as implemented in “GSVA” R package (Hänzelmann et al., 2013). ssGSEA applies gene signatures expressed by immune cell populations to individual high- and low-risk groups. In our study, we enrolled 28 immune cells of both innate and adaptive immunity. The correlations between levels of immune cells and genes involved in the diagnostic model were also assessed.

### Statistical Analysis

We performed D’Agostino and Pearson omnibus normality test to determine if datasets follow a normal distribution in each comparison. If the data passed the normality test, parametric tests were conducted (two-tailed unpaired t-tests, one-way ANOVA with Tukey’s correction for multiple comparisons, and Pearson correlation). If the data was not normally distributed, non-parametric tests were applied (Mann-Whitney-U test, one-way ANOVA using Kruskal-Wallis with Dunn’s correction for multiple comparisons, and Spearman correlation). The reported results apart from DEG analysis were all considered statistically significant at the 5% critical level (*p* < 0.05).

## RESULTS

### Data Series Screening and Study Design

To acquire qualified datasets, we systematically screened and obtained the peripheral blood microarray datasets of AR from GEO database (Figure 1). More detailed information can be found above in the Methods section. As shown in Figure 2, there are four parts in our research, including discovery, training, validation and further investigation phases. Baseline characteristics of recipients and donors involved in transplantation cohorts of our study were collected and illustrated in Table 1.

**TABLE 1:** Characteristics of three transplantation cohorts included in the current study.

Characteristics		GSE15296	GSE14346	GSE129166
Recipients	Age (year)	47.09 ± 14.55	11.70 ± 5.59	49.2 ± 13.8
	% Female	28.44	38.52	41
	HLA match	1.74 ± 1.38	2.43 ± 1.38	
	Immunosuppression			
	% Steroid free	51.38	54.1	13.7
	% Calcineurin inhibitors	91.74		92.3
	% Mycophenolic acid derivatives	82.57		84.6
Donors	Age (year)	38.97 ± 14.36	32.99 ± 12.05	49.8 ± 15.8
	% Female	44.95	50.82	52.2
	% Deceased donor	55.96	30.33	82

Values are demonstrated as means ± SD (Standard Deviation) or %. HLA, human leukocyte antigen.

### Identification of DEGs in AR

**FIGURE 3:**
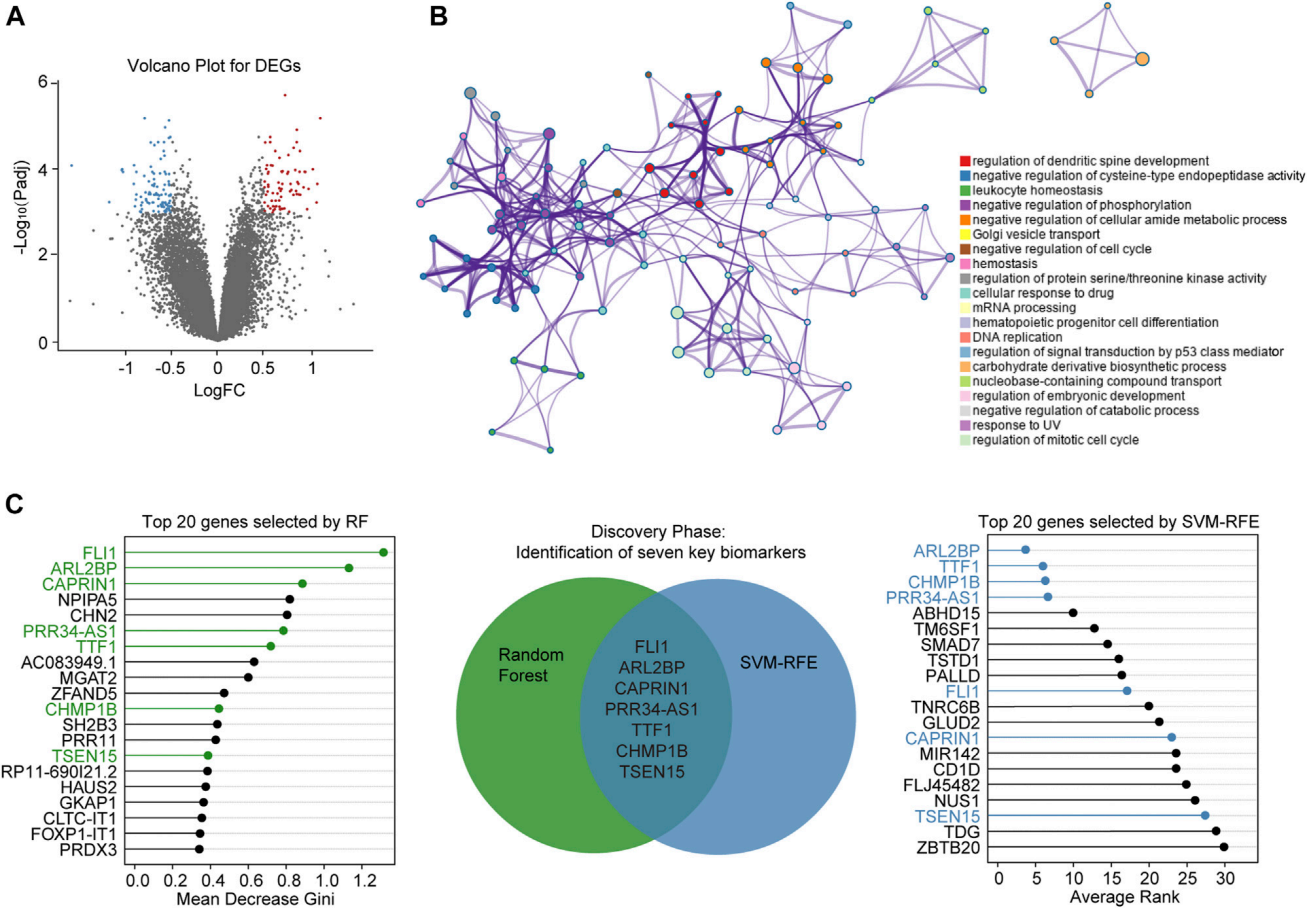
Screening of diagnostic biomarkers for AR **(A)** Volcano plot shows DEGs between AR and non-AR peripheral blood samples. Blue dots denote downregulated genes, and red dots denote upregulated genes **(B)** Network of top 20 enriched clusters, where each node represents one statistically significant term and terms with similarity of more than 0.3 are connected by edges **(C)** Lollipop chart shows top-ranked 20 genes ordered by Gini-importance through RF on the left side of the figure, while the same number of genes ranked by SVM-RFE are shown on the right side of the figure. Venn diagram for seven simultaneously top-ranked biomarkers is in the middle part, the green and blue circles represent DEGs selected by RF and SVM-RFE, respectively. DEGs: differentially expressed genes. FC: fold change. RF: random forest.

Following the screening, gene expression profiles of AR samples and non-AR samples from GSE15296 were selected for differential expression analysis. As a result, a total of 182 DEGs (78 upregulated and 104 downregulated) were identified for subsequent analyses (Figure 3A, Supplementary Table S1).

### Functional Enrichment Analysis

Metascape analysis showed the top 20 clusters of enriched biological processes (Figure 3B). Results manifested that the DEGs between AR and non-AR samples were significantly enriched in regulation of dendritic spine development, negative regulation of cysteine-type endopeptidase activity, leukocyte homeostasis and so on.

### Selection of Candidate Biomarkers

To identify key biomarkers for classifying AR and non-AR patients, machine learning methods including RF and SVM-RFE were adopted. Top-ranked 20 DEGs by each of the two algorithms with different phenotype-association measurements were appropriate for further analysis (Supplementary Table S2). After combining genes selected by the RF and SVM-RFE, seven biomarkers, including *TSEN15, CAPRIN1, PRR34-AS1, FLI1, TTF1, ARL2BP and CHMP1B,* were selected for the training phase (Figure 3C).

### Construction of the Diagnostic Model

**FIGURE 4:**
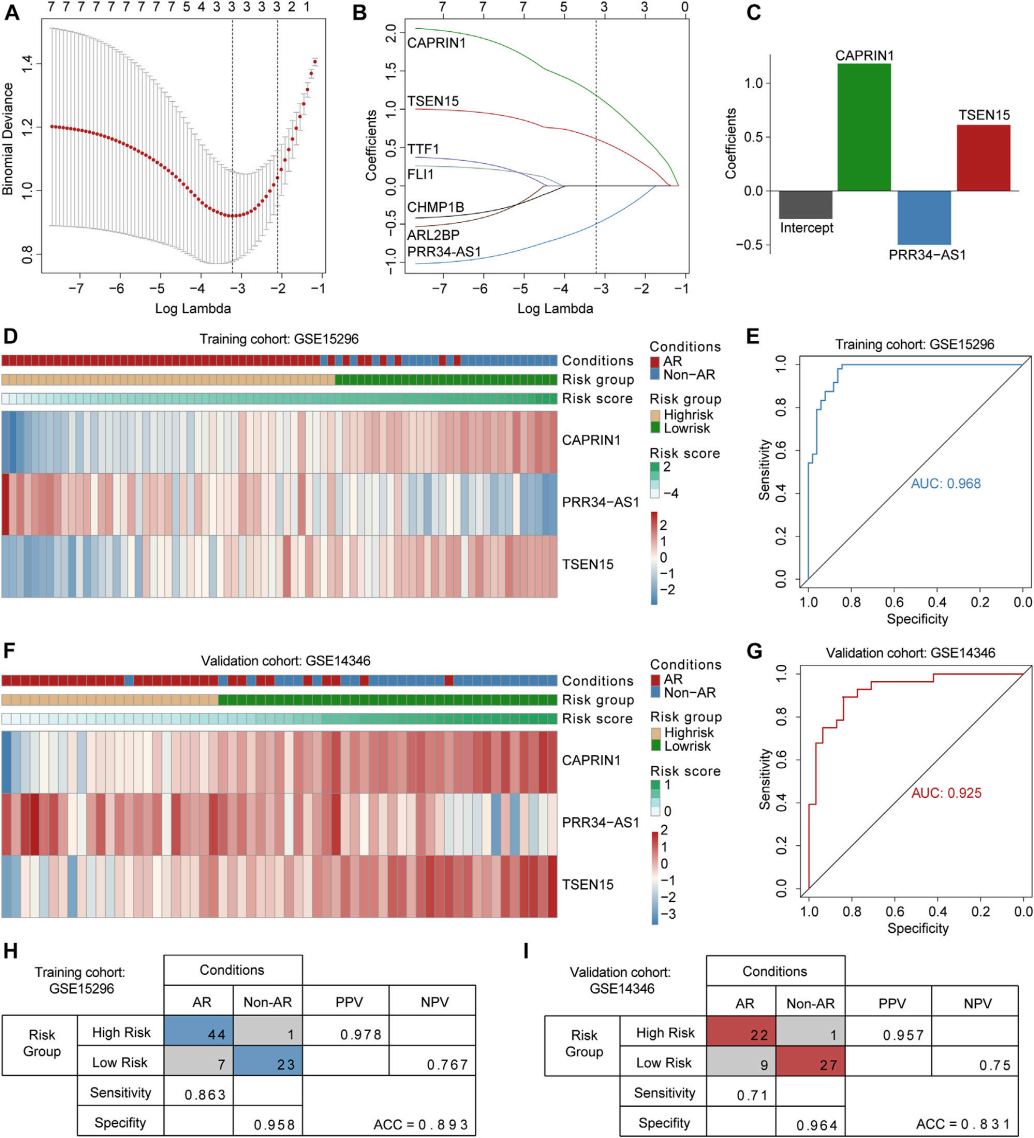
Construction and validation of the three gene-based diagnostic model **(A)** Selection of the tuning parameter in LASSO logistic regression analysis. Ten-fold cross-validation was utilized to calculate optimal lambda which leads to minimum mean cross-validation error **(B)** LASSO coefficient profiles, where each curve represents a DEG. Three DEGs were finally selected under the optimal lambda to construct a diagnostic model **(C)** The bar plot shows coefficients of three genes in the diagnostic model **(D and F)** Heatmaps for expression levels of three genes in the training and validation cohorts, patient annotations including disease status and risk score are also depicted **(E and G)** ROC curves for AR diagnosis prediction in the training and validation cohorts **(H and I)** Confusion matrices of binary results of the diagnostic model for training and validation cohorts. ROC curves: receiver operating characteristic curves. PPV: positive predictive value. NPV: negative predictive value.

The seven candidate genes screened in the discovery phase were applied to LASSO logistic regression to build the diagnostic model. Consequently, three optimal genes (*TSEN15, CAPRIN1* and *PRR34-AS1*) were employed to establish a diagnostic model (Figures 4A,B). The risk score formula was calculated as follows: risk score = -0.25938 + (0.61328 * expression level of *TSEN15*) + (-0.49935 * expression level of *PRR34-AS1*) + (1.18163 * expression level of *CAPRIN1*) (Figure 4C). The ROC curves indicated a high diagnostic power of the model (AUC = 0.968) with a specificity of 95.8% and a sensitivity of 86.3% (Figures 4E,H). Patients were classified into high- and low-risk groups based on a score of 0.3 as the optimal cutoff point (Figure 4D).

### External Validation of the Diagnostic Model

To verify the robustness of the three-gene diagnostic model, we employed an independent cohort GSE14346. Patients were divided into high-and low-risk groups relying on fixed formula and the same cutoff point obtained from training cohort (Figure 4F). Consistent with above findings, the ROC curves demonstrated a reliable diagnostic accuracy (AUC = 0.925) with a specificity of 96.4% and a sensitivity of 71% (Figures 4G,I). Results indicated that the diagnostic value of the model remained accurate and precise. Notably, the model demonstrated consistently high specificity in both training and validation cohorts.

### Gene Set Enrichment Analysis

**FIGURE 5:**
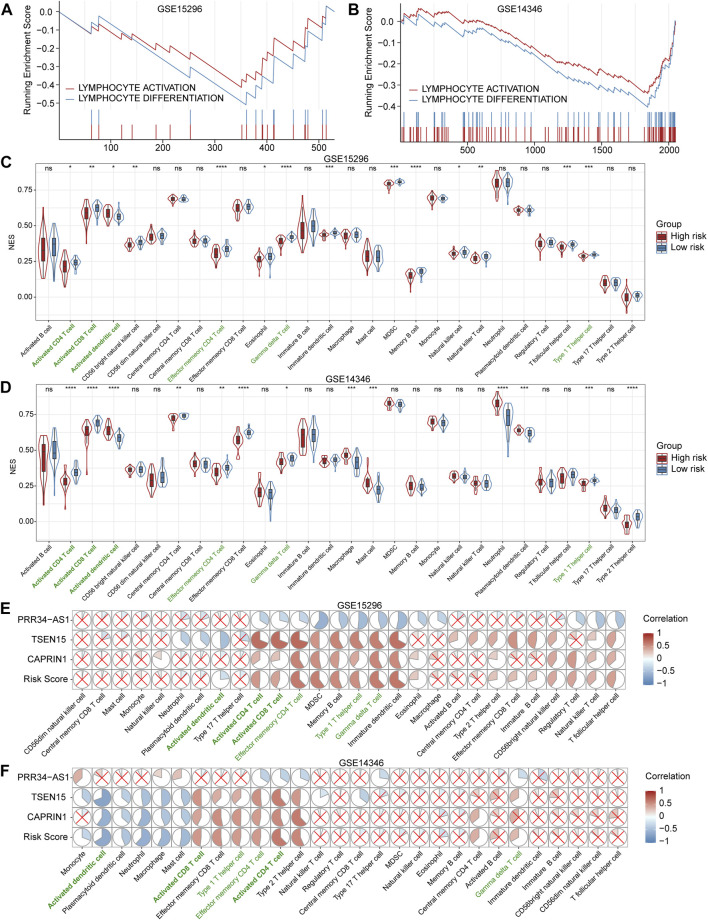
Gene set enrichment analysis (GSEA) and assessment of immune cells **(A and B)** GSEA plots show significantly enriched immune-related biological processes associated with the diagnostic model in the training (GSE15296) and validation (GSE14346) cohorts **(C and D)** Violin and box plots for comparisons of immune cell levels between high- and low-risk patients in the training and validation cohorts. Cells with statistically significant changes in both cohorts are labeled in green. *p < 0.05; **p < 0.01; ***p < 0.001; ****p < 0.0001; ns, not statistically significant **(E and F)** Correlation heatmaps between the expression levels of three genes and immune cell levels in the training and validation cohorts. The pie graphs are filled in proportion to the Spearman’s coefficient values, anti-clockwize for positive correlations (in red) and clockwise for negative correlations (in blue), the red crosses represent no statistically significant correlations (*p* > 0.05). NES: normalized enrichment score. MDSC: myeloid-derived suppressor cells.

GSEA was conducted to elucidate the potential biological processes occurring in high-risk patients compared to low-risk patients. As shown in Figures 5A,B, we found a significant aberrant reduction in the expression of genes associated with lymphocyte activation and differentiation for high-risk patients in both training and validation datasets. The above analyses may set the foundation for further exploring the molecular mechanisms of AR.

### Inference of Immune Cells in Peripheral Blood

We quantified 28 types of immune cells including the B cells, T cells, DCs, macrophages, natural killer cells and so on to investigate the composition of the peripheral blood by applying ssGSEA. As a result, the levels of activated CD4^+^ T cells, activated CD8^+^ T cells, effector memory CD4^+^ T cells, γδT cells and Th1 cells in the high-risk group were significantly lower than that in the low-risk group, while the levels of activated dendritic cells in the high-risk group were significantly higher than that in the low-risk group in both training and validation datasets (Figures 5C,D). Subsequently, we analyzed the correlation between these three genes involved in the diagnostic model and immune cells. Results showed that *PRR34-AS1* was negatively correlated with almost all immune cells, while *TSEN15* and *CAPRIN1* exhibited a similar positive correlation (Figures 5E,F). Briefly, these two genes were positively correlated with T cell subpopulations stably, consisting of Th1 cells, activated CD4^+^ and CD8^+^ T cells, etc. while negatively correlated with innate immune cells such as neutrophils, macrophages and dendritic cells. Also, the correlations of these three genes with immune cells showed the same trend as their coefficients in the diagnostic model.

### Assessment of TCMR and ABMR

**FIGURE 6:**
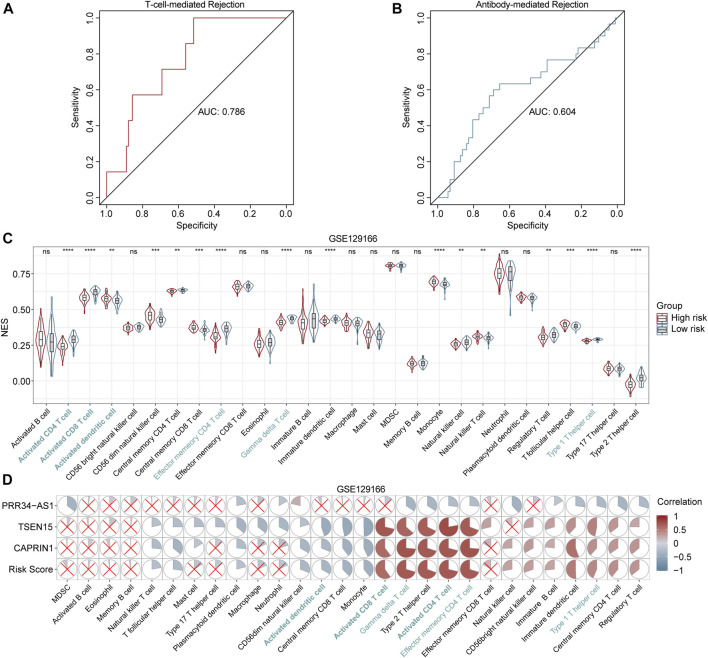
Performance of the diagnostic model in distinguishing TCMR and ABMR **(A and B)** ROC curves for TCMR and ABMR diagnosis in GSE129166 **(C)** Violin and box plots show the differences in immune cells between high- and low-risk patients in GSE129166. Common cells with different levels between two risk classes in all three cohorts (GSE15296, GSE14346 and GSE129166) are labeled in blue. *p < 0.05; **p < 0.01; ***p < 0.001; ****p < 0.0001; ns, not statistically significant **(D)** Correlation heatmaps between the immune cell levels and expression levels of three genes in GSE129166. Red and blue circles represent positive and negative correlations, respectively. Larger to smaller pie fill area indicates high to low correlation, no statistically significant correlations (*p* > 0.05) are represented by red crosses.

The aforementioned results revealed a notable correlation between the diagnostic model and adaptive immunity, especially T cell subsets. So we were curious if the model has a diagnostic power in classifying subtypes of immunologic rejection. Applying fixed formula and the same cutoff point, patients in GSE129166 were divided into high- and low-risk groups. As demonstrated in Figures 6A,B, results illustrated that the diagnostic model for AR showed a good ability in discriminating TCMR (AUC = 0.786), while a relatively poor ability in distinguishing ABMR (AUC = 0.604). Besides, consistent with previous results, six immune-cell subtypes with statistical significance in GSE15296 and GSE14346 showed the same apparent differences between high- and low-risk patients in GSE129166 (Figure 6C). Correlations between gene expression levels and immune cells also corresponded with above findings (Figure 6D). Those results further confirmed that our diagnostic model was associated with the immune status of renal transplant patients, especially with the effects of T cell subsets and DCs.

## Discussion

Acute rejection remains a major issue after kidney transplantation (Cravedi and Mannon, 2009; Li et al., 2012). In distinction to current clinical standards that depend on biopsy for AR diagnosis, early minimally invasive biomarkers would be a significant advance (Erpicum et al., 2017). In the present study, after systematically screened the datasets, two cohorts specifically focused on AR, which derived from multiple centers and combined more than 50 kidney transplantation patients were included for analysis. By applying machine learning methods and LASSO logistic regression, we identified and validated a three-gene model for AR diagnosis utilizing publicly peripheral blood gene expression data. Our diagnostic model showed a robust accuracy in both training (AUC = 0.968) and validation (AUC = 0.925) cohorts. Notably, the model demonstrated high sensitivity (0.958 in training cohort and 0.964 in validation cohort) which is more important for identifying cases as seen in our results. Hence, once classified into the high-risk group, the patient had a significantly high possibility to have ongoing AR and therefore need immediate further tests and treatment.

Intriguingly, according to the results of gene set enrichment analysis, lymphocyte activation and differentiation of high-risk patients were suppressed in peripheral blood. In agreement with this, we found a consistently lower amount of T cell subsets, such as activated CD4^+^ T cells, activated CD8^+^ T cells, effector memory CD4^+^ T cells and Th1 cells in peripheral blood of high-risk patients by using ssGSEA method. Those results indicated that our AR diagnostic model was tightly associated with T cells. In kidney transplantation, AR is predominantly T-cell mediated (Lee et al., 2014). Meanwhile, TCMR is composed mostly of acute TCMR and few of chronic active TCMR (Metter and Torrealba, 2020). Combined with our findings, we supposed that our AR model may also perform well in distinguishing TCMR. Therefore, another cohort derived from a multicenter study consisting of gene expression profiles of peripheral blood cells from kidney transplant patients with ABMR, TCMR or stable renal function was employed to verify the conjecture. Results illustrated a good performance for the assessment of TCMR (AUC = 0.786) as expected, which may indicate that high-risk patients assessed by the model can be diagnosed as TCMR and the robustness of the model was deeply verified.

Unlike kidney biopsy which reveals the levels of local immune cell infiltration (Sarwal et al., 2003), peripheral blood transcriptome reflects the overall immune status of kidney transplantation patients, which represents a more complex environment (Viklicky et al., 2020). In a previous study, downregulated genes in subclinical acute rejection were related to cytoskeleton organization, regulation of lymphocyte differentiation or cell death (Zhang et al., 2019), which is consistent with our results that genes involved in lymphocyte activation and differentiation were low expressed in the blood of high-risk patients with elevated tends to progress into AR. A similar phenomenon occurred in the assessment of immune cells, several subtypes of T cells were reduced in high-risk patients in all three cohorts, and a recent review also demonstrated the same decrease of T cell subtypes in peripheral blood of patients with allograft rejection (Mirzakhani et al., 2019). Our results suggested that the immune status in peripheral blood was so complicated, and the absence of increased transcription of immune response-related genes may support the speculation that immune cells migrated from the periphery to kidney allograft (Viklicky et al., 2020). These findings prompted us to pay more attention to investigate the systemic immune status of AR patients and its relationship with immune responses in renal allografts.

Among the three key genes involved in the current diagnostic model, *TSEN15* (tRNA splicing endonuclease subunit 15) is a protein-coding gene, catalyzing the removal of introns from tRNA precursors (Paushkin et al., 2004). tRNA splicing is highly conserved among vertebrates and is a fundamental process for cell growth and division. Researchers have reported that mutations in *TSEN15* cause neurogenetic disorders, including progressive microcephaly and pontocerebellar hypoplasia, which suggest its importance in brain development (Alazami et al., 2015; Breuss et al., 2016). Cytoplasmic activation/proliferation-associated protein-1 (*CAPRIN1*) belongs to a highly conserved protein family throughout vertebrate evolution (Zhang et al., 2018; Shi et al., 2019). *CAPRIN1* is closely associated with cancer cell cycle and cell proliferation, such as lymphocytes, human breast cancer cells, cervical cancer Hela cells (Grill et al., 2004). It has been reported that suppression of *CAPRIN1* leads to a slower proliferation rate and prolonged G1 phase of the cell cycle (Wang et al., 2005). In brief, *TSEN15* and *CAPRIN1* play vital roles in cell cycle and proliferation. However, their roles in the episodes of AR remain unclear. By analyzing the correlation between expression levels of these two genes and immune cells, we found that *TSEN15* and *CAPRIN1* were both negatively correlated with innate immune cells such as neutrophils, macrophages, dendritic cells, while positively correlated with T cell subpopulations, including Th1 cells, activated T cells, etc. Therefore, high-risk patients had lower expression levels of *TSEN15* and *CAPRIN1,* which may suppress CD4^+^T, CD8^+^T cells or enhance macrophages, neutrophils to participate in the occurrence of AR. *PRR34-AS1* (PRR34 antisense RNA 1) is an RNA gene affiliated with the long noncoding RNA (lncRNA) class (Zhao et al., 2017; Tao et al., 2019). It has been reported that *PRR34-AS1* is closely associated with the early tumor recurrence in bile duct cancer (Fang et al., 2020), and it has been proved to potentially relieve ischemic reperfusion injury after total knee arthroplasty in mice by diminishing apoptosis and enhancing cell proliferation of chondrocyte *in vitro* (Fang et al., 2020). In our research, the expression level of *PRR34-AS1* was negatively correlated with nearly all immune cells and its overexpression indicated a higher risk to be diagnosed as AR patients, which may suggest a novel insight into the relationship between gene and cells. Further research is urgently needed to verify those assumptions and clarify the complex interactions between genes and immune cells in peripheral blood.

Over the last decades, there has been a rapid increase in the number of non-invasive biomarkers for predicting acute rejection after kidney transplantation, including gene expression data (Li et al., 2012; O'Callaghan and Knight, 2019). Many of these multiple-gene panels have provided reasonable acute rejection response prediction, with accuracy ranging from 80% to 90% derived from specific cohorts (Einecke et al., 2010; Kurian et al., 2014; Shaw et al., 2020). However, the transferability and reproducibility of these biomarkers or gene-based panels remain limited. Recently, a study reported that a set of gene signature developed based on a single study does not appear to provide adequate prediction in other independent cohorts with reduced predictability of less than 50% (Cao et al., 2020). Besides, diagnostic models developed in a few studies consisted of dozens or hundreds of genes, which greatly limited their clinical applications (Friedewald et al., 2019; Cao et al., 2020). In the current research, we applied machine learning methods and LASSO logistic regression to identify a novel gene-based diagnostic model consisting of only three genes, results indicated consistently high accuracy and sensitivity of the model with AUC values for AR higher than 0.9 in both training and validation cohorts. Moreover, the diagnostic model was more related to T cells and performed well in distinguishing TCMR. However, there are still some limitations to this study. It was serendipitous that all three datasets were of the same platform and external validation of the gene signature is needed. Further prospective studies with larger cohorts in more centers are required to validate the accuracy and reproducibility of this model. Besides, the precise biological mechanisms underlying these three genes are still unclear in AR progression and needed to be more emphasized in functional experiments. Although an interesting phenomenon was detected, relationships between overall immune status and kidney allograft immune response were necessary to be clarified in the future. Despite those limitations, our study proved that peripheral blood biomarkers have the potential to alert physicians during the early stages of rejection, and the three-gene diagnostic model could be conveniently used as a non-invasive peripheral test for renal transplant patients in clinical practice.

In summary, a novel diagnostic model for AR consisting of only three genes was developed and validated for post-transplant patients. Measuring the expression levels of these three genes may provide a cost-effective and accurate individualized method for clinical monitoring and diagnosis in AR. Besides, our model was closely related to the immune status of renal transplant patients in peripheral blood, which provides insights for further investigating potential mechanisms and therapeutic targets for AR.

## Data Availability

The original contributions presented in the study are included in the article/Supplementary Material, further inquiries can be directed to the corresponding author.

## Abbreviations

ABMR, antibody-mediated rejection; AR, acute rejection; DEGs, differentially expressed genes; GEO, Gene Expression Omnibus; GSEA, gene set enrichment analysis; LASSO, least absolute shrinkage and selection operator; RF, random forest; ssGSEA, single sample gene set enrichment analysis; STA, stable; ROC, receiver operating characteristic; SVM-RFE, support vector machine-recursive feature elimination; TCMR, T-cell mediated rejection.
