# Supplementary material for: A Three-Gene Peripheral Blood Potential Diagnosis Signature for Acute Rejection in Renal Transplantation
Source: Front Mol Biosci. 2021 May 4;8:661661. doi: 10.3389/fmolb.2021.661661 (PMC8129004; doi:10.3389/fmolb.2021.661661)
Supplement: Supplementary file 1 [file DataSheet1.docx]

**SUPPLEMENTARY MATERIAL**

**Supplementary Table 1:** One hundred and eighty-two differentially expressed genes between AR and non-AR peripheral blood samples. AR: acute rejection. FC: fold change.

| Gene | Log FC | Adjusted P-value |
| --- | --- | --- |
| PRR34-AS1 | 0.72085859 | 1.71E-06 |
| AC083949.1 | 1.097903177 | 5.93E-06 |
| FLI1 | -0.783252973 | 5.93E-06 |
| CAPRIN1 | -0.522539253 | 6.68E-06 |
| MGAT2 | -0.56358978 | 9.70E-06 |
| RUSC1-AS1 | 0.847206282 | 1.10E-05 |
| PRKAG2 | -0.508490825 | 1.64E-05 |
| PRRC2C | 0.561633588 | 1.64E-05 |
| CHN2 | -0.523814668 | 1.72E-05 |
| FOXP1-IT1 | 0.845466808 | 2.17E-05 |
| NUS1P3 | -0.638852073 | 2.17E-05 |
| INSIG1 | -0.575587103 | 2.17E-05 |
| TTF1 | 0.561096627 | 2.17E-05 |
| TSTD1 | 0.507447748 | 2.21E-05 |
| ARL2BP | -0.717409148 | 2.21E-05 |
| ZFAND5 | -0.678099136 | 2.54E-05 |
| TAF15 | 0.81256395 | 2.93E-05 |
| SH2B3 | -0.578893072 | 3.50E-05 |
| NKTR | 0.616377914 | 3.50E-05 |
| BD495725 | 0.880258679 | 3.50E-05 |
| ANKRD13D | 0.756248144 | 4.34E-05 |
| CNOT8 | -0.570877 | 4.50E-05 |
| TRMT10B | 0.660291256 | 5.08E-05 |
| MAPKAPK5 | -0.611230792 | 5.36E-05 |
| UVSSA | 0.526846331 | 7.54E-05 |
| RUNX1-IT1 | 0.570331841 | 7.54E-05 |
| KLF4 | -0.896679006 | 7.54E-05 |
| CXCL8 | -1.565379899 | 7.69E-05 |
| ITPKB | 0.585967393 | 7.72E-05 |
| DLAT | -0.716832506 | 8.17E-05 |
| SLC25A16 | 0.518261515 | 9.20E-05 |
| SRGAP2B | 1.017978871 | 9.36E-05 |
| ATF1 | -1.024003853 | 9.36E-05 |
| RFWD3 | 0.829301967 | 9.78E-05 |
| SGPP1 | -1.029437331 | 9.78E-05 |
| LOC101060275 | 0.693408719 | 0.000103817 |
| MIR142 | 0.683411259 | 0.000106219 |
| AK025288 | 0.929994715 | 0.000107767 |
| LOC100631377 | 0.875168886 | 0.000107767 |
| RAB5A | -0.568592378 | 0.000107767 |
| PRDX3 | -0.700514603 | 0.000108385 |
| PRNP | -0.871804554 | 0.000108385 |
| SRRM2 | 0.541955952 | 0.000108385 |
| LOC100131541 | 0.937065349 | 0.000108385 |
| RP11-690I21.2 | 0.754037486 | 0.000108385 |
| SH2D1B | -1.01498659 | 0.000108385 |
| SNORA76C | 0.687937611 | 0.000108385 |
| SIAH1 | -0.619040363 | 0.00011775 |
| DHX36 | 0.536304214 | 0.000118857 |
| DHRS4-AS1 | -0.683384949 | 0.000118857 |
| LOC102725188 | 0.74483854 | 0.000119873 |
| TSEN15 | -0.521271402 | 0.000121762 |
| FAM220A | -0.556200071 | 0.000121762 |
| NUS1 | -0.78764533 | 0.000134771 |
| MYCL | -0.682135633 | 0.000161071 |
| SPRTN | -0.525831297 | 0.000181797 |
| CHMP1B | -0.738342245 | 0.000181797 |
| ZBTB20 | 0.502483367 | 0.000182829 |
| GCNT7 | 0.853006381 | 0.000188411 |
| SSBP3-AS1 | 0.649687787 | 0.00019252 |
| LOC102724250 | 0.864112308 | 0.000205904 |
| FAM185A | 0.778861473 | 0.000207709 |
| DSERG1 | 0.690525373 | 0.000207709 |
| BET1 | -0.899652604 | 0.000207709 |
| PLCB1 | -0.678542927 | 0.000207709 |
| LOC142937 | 1.065687085 | 0.000207709 |
| ATP6AP2 | -0.521878095 | 0.000210104 |
| ARFGAP3 | -0.567763668 | 0.000216257 |
| HIST1H4J | 0.539781674 | 0.000216257 |
| FAM206A | -0.520027525 | 0.000222146 |
| SMAD7 | -0.700309965 | 0.000222146 |
| BZW1 | -0.684578253 | 0.000230723 |
| TMEM63A | 0.540734514 | 0.000241983 |
| GLUD2 | -0.650042252 | 0.000241983 |
| AY940074 | 0.918746298 | 0.000250252 |
| ZNF562 | 0.500933898 | 0.000250696 |
| ALMS1 | 0.658258995 | 0.000261185 |
| CLTC-IT1 | 0.972042855 | 0.000271271 |
| AX747652 | 0.682013194 | 0.000271271 |
| MTM1 | -0.579705198 | 0.000285992 |
| CCZ1B | 0.787658648 | 0.000287812 |
| LOC100289058 | 0.671993534 | 0.00030341 |
| RP11-138A9.1 | 0.838335666 | 0.000303761 |
| STAG3L3 | 0.774219151 | 0.000310521 |
| NCLN | 0.533476251 | 0.00031388 |
| FYTTD1 | -0.731396801 | 0.000327791 |
| TM6SF1 | -0.773255006 | 0.000328628 |
| UPRT | -0.728414215 | 0.000333516 |
| MICAL1 | 0.588908317 | 0.000343361 |
| HHEX | -0.658989857 | 0.00034658 |
| GTF2H3 | 0.639363949 | 0.000349971 |
| SERP1 | -0.720475963 | 0.000360664 |
| ADSS | -0.557680673 | 0.000360664 |
| AASDHPPT | -0.735605462 | 0.000360664 |
| AGAP4 | 0.845235151 | 0.000360664 |
| SLC25A43 | -0.738042593 | 0.000360664 |
| FAM174A | -0.871581782 | 0.000367607 |
| FZD1 | -0.503920117 | 0.000367607 |
| LOC732360 | 0.868708979 | 0.000367607 |
| SNX30 | -0.652973538 | 0.000372715 |
| LOC727820 | 0.516637452 | 0.000372842 |
| CLINT1 | -0.640383135 | 0.000372842 |
| TAB2 | -0.674230858 | 0.000372842 |
| BC048103 | 0.88350205 | 0.000372842 |
| DNAJB6 | -0.680313034 | 0.000372842 |
| IER5 | -0.603756662 | 0.000372842 |
| LOC100272216 | 0.810802375 | 0.000376241 |
| CD1D | -0.505972252 | 0.000390062 |
| GTF2E1 | -0.629418085 | 0.000391959 |
| RCAN1 | -0.521564388 | 0.000391959 |
| SGK1 | -0.823963686 | 0.000417634 |
| LOC340085 | 0.677907771 | 0.000419389 |
| SLC39A6 | -0.63609785 | 0.00046634 |
| DNAJC10 | -0.768021875 | 0.00047303 |
| PMAIP1 | -0.8767584 | 0.000486798 |
| ZNF721 | 0.657174714 | 0.000508481 |
| SELT | -0.766389741 | 0.00050927 |
| LOC100289333 | 0.62005194 | 0.000513088 |
| AX748417 | 0.578322561 | 0.000514789 |
| UBTD2 | -0.512892144 | 0.000518439 |
| LOC646014 | 0.577256527 | 0.000519862 |
| SMIM15 | -1.162534917 | 0.000555797 |
| UBL3 | -0.69719367 | 0.000566623 |
| EIF4E | -0.561097644 | 0.000566825 |
| RPA3OS | -0.690972764 | 0.000567318 |
| AP1S2 | -0.750605825 | 0.000567318 |
| LOC102724587 | 1.057072245 | 0.000567318 |
| DBT | 0.501003674 | 0.000567318 |
| SLBP | -0.584416513 | 0.000567318 |
| TBC1D9 | -0.809815141 | 0.000567785 |
| NUDT21 | -0.593193823 | 0.000567785 |
| HMGCR | -0.522383359 | 0.000585663 |
| YAF2 | -0.561843388 | 0.000585663 |
| ADO | -0.520452136 | 0.000585663 |
| COG6 | -0.733706488 | 0.000595927 |
| PSMD10 | -0.60944137 | 0.00063319 |
| PRMT9 | -0.55296183 | 0.0006373 |
| LYSMD2 | -0.545100985 | 0.000639371 |
| AGGF1 | -0.546339774 | 0.00064569 |
| KIFAP3 | -0.590753423 | 0.000648526 |
| CCNG2 | -0.821028361 | 0.000661479 |
| PDE4B | -0.593805399 | 0.000685168 |
| TRAPPC11 | -0.534274891 | 0.000685663 |
| TADA1 | -0.604478696 | 0.000686265 |
| FLJ45482 | 0.538068195 | 0.000686265 |
| LMBRD1 | -0.637807253 | 0.000686265 |
| ZBTB1 | -0.886495771 | 0.000695478 |
| FBXW12 | 0.626429718 | 0.000695478 |
| ELMSAN1 | -0.514707817 | 0.000696962 |
| GOLPH3 | -0.500021997 | 0.000696962 |
| KIAA0754 | 0.592307899 | 0.000697069 |
| GKAP1 | 0.617798374 | 0.000707821 |
| DLGAP4 | 0.660264774 | 0.000711117 |
| PALLD | -0.597967203 | 0.000713723 |
| SYPL1 | -0.576459547 | 0.000727186 |
| C8orf44 | 0.712815329 | 0.000782827 |
| LOC101929889 | 0.56203673 | 0.000783138 |
| CXCL6 | -0.544214998 | 0.000795226 |
| MZT2B | 0.681041361 | 0.000801113 |
| LOC286437 | 0.69390547 | 0.000801113 |
| SDE2 | -0.544771006 | 0.000801113 |
| HDAC2 | -0.523310993 | 0.000801113 |
| ABHD15 | -0.529893656 | 0.000801113 |
| FTX | 0.956945096 | 0.000818212 |
| CDK13 | 0.609077279 | 0.000819014 |
| ZNF160 | 0.665284423 | 0.000819014 |
| CAAP1 | -0.81220179 | 0.000819014 |
| FLJ38717 | 0.671283783 | 0.000823246 |
| PRR11 | 0.596098617 | 0.000867497 |
| MAP2K4 | -0.517713376 | 0.000867497 |
| LIG4 | -0.55757791 | 0.000867497 |
| PRKAR1A | -0.550336528 | 0.00090399 |
| RFK | -0.688065026 | 0.000904552 |
| TNRC6B | 0.504032435 | 0.000904552 |
| GMCL1 | -0.570852555 | 0.000911144 |
| THAP1 | -0.830865894 | 0.00092721 |
| ZNF304 | -0.633357114 | 0.000936231 |
| ZC3H7B | 0.764058993 | 0.00093633 |
| SLC25A32 | -0.82178344 | 0.00095269 |
| CRNKL1 | -0.506082851 | 0.000976508 |
| HAUS2 | 0.587091004 | 0.000985538 |
| FDX1 | -0.560641905 | 0.000993453 |

**Supplementary Table 2**: Top-ranked 20 genes by RF and SVM-RFE. RF: random forest. SVM-RFE: support vector machine - recursive feature elimination.

| RF | | SVM-RFE | |
| --- | --- | --- | --- |
| Gene | Gini-Importance | Gene | Average-Rank |
| FLI1 | 1.321204 | ARL2BP | 6.1 |
| ARL2BP | 1.138744 | PRR34-AS1 | 6.2 |
| CAPRIN1 | 0.893024 | TTF1 | 8.3 |
| LOC101060275 | 0.826387 | SMAD7 | 8.5 |
| CHN2 | 0.812176 | GLUD2 | 8.9 |
| PRR34-AS1 | 0.79266 | TM6SF1 | 10.4 |
| TTF1 | 0.725916 | CHMP1B | 13.4 |
| AC083949.1 | 0.637915 | ABHD15 | 14.9 |
| MGAT2 | 0.607133 | FLJ45482 | 16.8 |
| ZFAND5 | 0.479509 | PALLD | 17.6 |
| CHMP1B | 0.452032 | CD1D | 20.2 |
| SH2B3 | 0.443685 | FLI1 | 21.2 |
| PRR11 | 0.43515 | TSEN15 | 22.9 |
| TSEN15 | 0.394608 | LOC732360 | 23.7 |
| RP11-690I21.2 | 0.391687 | ZBTB20 | 24.8 |
| HAUS2 | 0.382299 | NUS1 | 25 |
| GKAP1 | 0.371022 | TSTD1 | 26.1 |
| CLTC-IT1 | 0.36176 | CAPRIN1 | 30.5 |
| FOXP1-IT1 | 0.352341 | TNRC6B | 30.9 |
| PRDX3 | 0.348343 | PSMD10 | 32.2 |
